# Predictors of Metabolic Dysfunction-Associated Steatotic Liver Diseases (MALSD) in Vietnamese Population

**DOI:** 10.21203/rs.3.rs-8130849/v1

**Published:** 2025-12-04

**Authors:** Anh Gia Pham, An Vu-Khanh Le, Yen Thi-Hai Pham, Lanh Sy Nguyen, Huong Lan Nguyen, Nghia Chinh Quach, Thau Manh Cao, Hanh Thi Nguyen, Xuan Thanh Thai, Anh Tuan Luong, Thuy Thi Pham, Hai Duy Vu, Hai Thanh Tran, Lan Thi Le, Hai Vu, Giang Binh Tran, Hung N. Luu

**Affiliations:** Viet Duc University Hospital; Viet Duc University Hospital; University of Pittsburgh Medical Center; Viet Duc University Hospital; Viet Duc University Hospital; Viet Duc University Hospital; Viet Duc University Hospital; Viet Duc University Hospital; Viet Duc University Hospital; Viet Duc University Hospital; Hanoi University of Science and Technology; Hanoi University of Science and Technology; Hanoi University of Science and Technology; Hanoi University of Science and Technology; Hanoi University of Science and Technology; Viet Duc University Hospital; University of Pittsburgh Medical Center

**Keywords:** Predictors, MASLD, NAFLD, hepatocellular carcinoma, Vietnam

## Abstract

**Background and Aims.:**

Metabolic dysfunction-associated steatotic liver disease (MASLD) comprises a spectrum of liver diseases from simple steatosis, non-alcoholic steatohepatitis (NASH) which progresses to fibrosis and compensated cirrhosis. Worldwide, MASLD significantly contributes to the rising incidence of liver cancer. Lille is known about its predictors in Asian population and particularly among Vietnamese.

**Approach and Results.:**

We determine the predictors of MASLD in Vietnamese population using a hospital-based case-control study of 100 MASLD patients and 119 healthy controls. Unconditional logistic regression model was used to calculate odds ratios (ORs) and respective 95% confidence intervals (CIs) for MASLD in relation to potential predictors. Overall, body mass index (BMI) (OR_> 23 vs. ≤23 kg/m2_=1.93, 95% CI: 1.00–3.72), diabetes (OR = 4.46, 95% CI: 1.16–17.12); AST (OR_≥40 vs. <40_=4.18, 95% CI: 1.54–11.33), GGT (OR_≥55738 vs. <55738_=5.20, 95% CI: 2.39–11.33), international normalized ratio (INR) (OR_> 1.0 vs. ≤1.0_=2.18, 95% CI: 1.15–4.14) kPA (_>8.2 vs. ≤8.2_=11.14, 95% CI: 1.29–96.05) and CAP score (OR_≤ 257.1 vs. >257.1_=30.21, 95% CI: 10.07–90.64) were the predictors for MASLD. Other important clinical factors, including cardiometabolic condition, platelet, ALT, albumin, cholesterol or triglyceride were not associated with risk of MALSD.

**Conclusions.:**

Besides clinical factors such as BMI, diabetes, AST, and GGT, liver stiffness measurement (i.e., kPA and CAP score) played important roles as predictors for MALSD in Vietnamese population. Our findings, for the first time, provided evidence for risk stratification and treatment management of MALSD in Vietnamese population.

## INTRODUCTION

In 2024, liver cancer was ranked 6th most common cancer and 3rd leading cause of cancer death worldwide, with annually close to 900,000 new cases and approximately 800,000 deaths worldwide.^1^ In Vietnam, primary liver cancer is the leading cancer in both incidence and mortality, accounting for 15.4% and 22.1% of total cancer, respectively.^2^ Hepatocellular carcinoma (HCC) is the most common histological subtype, accounting for 90–95% of all primary liver cancer cases in Vietnam.^3^ Established major risk factors for HCC are chronic infection with hepatitis B (HBV) and hepatitis C virus (HCV), excessive alcohol use, and to a lesser extent, dietary exposure to aflatoxin in certain regions.^3^ Given the diminishing role of HBV (due to universal vaccination program) and HCV (available curative therapy), metabolic dysfunction-associated steatotic liver disease (or MASLD),^4^ a nomenclature of non-alcoholic fatty liver disease (or NAFLD), has emerged as the most important risk factor for HCC.

MASLD/NAFLD (both terms being interchangeable) comprises a spectrum of liver diseases from simple steatosis, non-alcoholic steatohepatitis (NASH) which progresses to fibrosis and compensated cirrhosis. Compensated cirrhosis would further develop to HCC and decompensated cirrhosis that requires liver transplantation or leads to death (hereafter referred as “end-stage liver diseases”).^5^ The prevalence of MASLD is about 25% of adult population worldwide^6^ and close to 34% in Asia during 2012–2017 period.^7^ MASLD significantly contributes to the rising incidence of liver cancer in the U.S and worldwide. It is estimated that the cumulative incidence of HCC is around 2.4–12.8% among patients with NASH with advanced fibrosis or compensated cirrhosis over 3–7 years.^8^ MASLD is the second, and will soon take over viral hepatitis as the most common indication for HCC/liver transplantation in the U.S. and worldwide.^9^ It is therefore an urgent and unmet need to identify predictors of MASLD.

MASLD is found to be associated with metabolic syndrome (i.e., obesity, insulin resistance, type 2 diabetes mellitus-T2DM), and cardiovascular complications (i.e., dyslipidemia and hypertension).^10,11^ Also, while biopsy is considered a gold standard for MASLD diagnosis, its major drawback is the invasives. Different non-invasive modalities have been developed to compensate the biopsy, including diagnostic imaging or clinical-based fibrosis scores. Specifically, liver stiffness measurement (LSM) by vibration-controlled transient elastography (VCTE) has recently applied and validated, serving as a modality to reflect the degree of liver fibrosis and predict HCC, portal hypertension, and varices.^12^ In addition, five laboratory tests, considered non-invasive biomarkers have been established markers for fibrosis diagnosis, including 1) The aspartate aminotransferase (AST)-to-platelet ratio index (APRI)^13^ 2) The AST/ALT ratio (alanine aminotransferase) ratio; 3) the (fibrosis 4-index) FIB-4^14^; 4) the NAFLD fibrosis score (NFS)^15^; and 5) the BARD score^16^ where BMI, AST/ALT ratio and diabetes are included in this score. In a recent meta-analysis to determine the diagnostic accuracy of APRI (35 studies), FIB-4 (37 studies), BARD score (30 studies), NSF (41 studies), VCTE using FibroScan (19 studies), SWE (4 studies), MRE (5 studies), Xiao et al.^17^ reported that among non-invasive biomarkers, NFS and FIB-4 offer optimal diagnostic accuracy. Recently, two scores, called the Agile 3+-serving-serving for the diagnosis of advanced fibrosis and the Agile 4-serving for the diagnosis of cirrhosis for the diagnosis of advanced fibrosis and the Agile 4-serving for the diagnosis of cirrhosis, were proposed by combining LSM and several clinical variables, including platelet count, AST, diabetes, age, and sex).^18,19^ While such non-invasive modalities have been examined in MASLD patients of Caucasian population, little efforts have been made in Asian population and none in Vietnamese population.

To fill this gap of knowledge, we determined the potential predictors of MALSD in Vietnamese population in a case-control study of 100 MASLD patients and 119 healthy controls.

## METHODS

### Study Population

The current analysis used data from a hospital-based case-control study of 100 MASLD patients and 119 healthy controls who were recruited at the Viet-Duc University Hospital between November 1, 2020, through December 31, 2024. All study participants agreed to provide written informed consent before participating in the current study. Our study was approved by the Institutional Review Boards (IRBs) of the participating institutions, including the Viet-Duc University Hospital and the University of Pittsburgh. All research was conducted in accordance with both the Declarations of Helsinki and Istanbul

### Recruitment of MASLD Patients

Patients with MASLD were recruited at the Department of Diagnostic Imaging, Department of Surgical Oncology and Department of General Examination of the Viet-Duc University Hospital November 1, 2020, through December 31, 2024. Physical exam was performed and clinical and laboratory tests were conducted to confirm the inclusion/exclusion criteria. Inclusion criteria^4^: 1) Age 40–79 years and 2) non-alcoholic consumption; and 3) steatosis confirmed using the international criteria^20^ (i.e., CAP score ≥ 237 (steatosis) or kPa ≥ 5.5 (fibrosis); and 3) at least one out of five cardiometabolic criteria (i.e., obesity/overweight, diabetes, hypertension or hyperlipidemia) as follow: [1] BMI > 23 kg/m^2^ or waist-circumference > 94 cm (Male) or 80cm (Female); or [2] Diabetes diagnosis (i.e., Fasting serum glucose ≥5.6 mml./L (100mg/dL) OR 2-hour post-load glucose levels ≥ 7.8mmol/L (≥140mg/dL) OR HbA1c≥5.7% (39mmol/L) OR Type 2 diabetes OR Treatment for type 2 diabetes); or [3] hypertension or CVD diagnosis (i.e., Blood pressure ≥135/85mmHg OR Specific antihypertensive drug treatment); or [4] Plasma triglycerides ≥1.70 mmol/L (150mg/dL) OR Lipid lowering treatment; or [5] Plasma HDL-cholesterol ≤ 1.0mmol/L (40mg/dL) (Male) and ≤ 1.3mmol/L (50mg/dL) (Female) OR Lipid lowering treatment. Exclusion criteria were (1) alcoholic liver disease or consumption of alcohol > 14 drinks/week for women or > 21 drinks/week for men in the past 12 months; (2) other chronic liver diseases including autoimmune hepatitis, biliary cirrhosis, hemochromatosis and Wilson’s disease; (3) positive serological test for HBsAg, HBV DNA, antibodies to HCV or HCV RNA; (4) the presence of HCC), (5) history of liver transplantation and/or decompensated cirrhosis; (6) cancer under active treatment; (7) uncontrolled psychiatric syndrome; (8) serious co-morbidities (e.g., unstable cardiovascular disease and dialysis); and (9) use of antibiotics for ≥7 days within the past 60 days.

### Recruitment of Healthy Control Subjects

Healthy control subjects were recruited from the same three departments at the Viet-Duc University Hospital using the following criteria: individual who were 40–79 years of age, BMI 20–<35 kg/m^2^, and no history of MASLD, diabetes, or any conditions listed in the Inclusion/Exclusion criteria above and those who agreed to participate in the study.

### Exposure Information Collection

A structured questionnaire was used by a trained interviewer to collect exposure information from the study participants at baseline. The structured questionnaire included the following components: 1) sociodemographic factors; 2) lifetime tobacco and alcohol use; 3) body height and weight; 4) dietary habits; 5) medical history; 6) family history of cancer and 7) occupational exposures. Medical information from the medical records were extracted by the trained medical extractors.

### Liver stiffness measurement (LSM)

The liver stiffness measurement was performed using FibroScan model 430 (EchoSens, Paris, France), following instructions and training provided by manufacturer^21^. Briefly, measurements was performed on the right lobe of the liver through intercostal spaces with the patient lying in dorsal decubitus with the right arm in maximal abduction. The tip of the probe transducer was covered with coupling gel and placed on the skin, between the ribs at the level of the right lobe of the liver. The operator, assisted by ultrasound time-motion and A-mode images provided by the system, located a portion of the liver that is at least 6cm thick and free of large vascular structures. Once the area of measurement is located, the operator pressed the probe button to begin an acquisition. The measurement depth will be between 25 and 45mm. Ten successful acquisitions were performed on each patient. The median value was represented the liver elastic modulus. Evaluation was only be performed on 10 successful acquisitions in which the reliability was considered as at least 60% success rate. The success rate was calculated as the number of successful measurements divided by the total number of measurements. The liver stiffness was expressed in kiloPascal (kPa).

### Non-invasive fibrosis scores

Five non-invasive fibrosis scores were considered in the current study. (1) The APRI was calculated as AST (/upper limit of normal)/platelet count (×10^9^/L)×100.^13^ (2) The AST/ALT ratio. (3) The FIB-4 was calculated as age (year)×AST (U/L)/platelet count (×10^9^/L)×[ALT(U/L)]^1/2^.^14^ The cut-off of FIB-4 for NAFLD patients was be adopted (i.e., 0.97 for F0–2 and 1.98 for F3–4 in Caucasian population)^22^ and the FIB-4 was validated in Japanese population (i.e., 1.13 for F0–2 and 3.17 for F3–4)^23^. (4) The NFS score is calculated as: −1.675 + 0.037×age (years) + 0.094×BMI(kg/m^2^) + 1.13×impaired fasting glycemia (IFG)/diabetes (yes = 1, no = 0) + 0.99×AST/ALT ratio−0.013×platelet (×10^9^/L)−0.66×albumin (g/dL).^15^ And (5) the BARD score is the weighted sum of three variables (BMI ≥ 28 = 1 point, AST/ALT ratio ≥ 0.8 = 2 points, diabetes = 1 point).^16^

### Statistical Analysis

Means and standard deviation (SD) were calculated for continuous variables whereas counts and proportions were computed for categorical variables. To compare the distributions of continuous and categorical variables between cases and controls, *t*-test or analysis of variance (ANOVA) and *χ*^*2*^ tests were applied, respectively.

Unconditional logistic regression models were used to determine the association between potential predictors and risk of MASLD in the current study, producing odds ratios (ORs) and respective 95% CIs. Potential predictors, including socio-economic factors and clinical features were included in the logistic regression models such as age, sex, BMI, diabetes status (yes vs. no), cardiometabolic condition, platelet (> 200 vs. ≤200G/L), INR (≤ 1.0 vs. >1.0), AST (< 40 vs. ≥40U/L), ALT (< 41 vs. ≥41U/L), GGT (< 55738 vs. ≥55738U/L), albumin (35–50, < 35 and > 50g/L), cholesterol (≤ 5.2 vs. >5.2mmol/L), triglyceride (< 1.7 vs. ≥1.7mmol/L), HDL (> 0.9 vs. ≤0.9mmol/L), LDL (< 4.0 vs. ≥4.0mmol/L), HbA1c (< 5.7% vs. ≥5.7%), FIB-4 (< 1.3 vs. ≥1.3), kPA (≤ 8.2 vs. >8.2), CAP score (≤ 257.1 vs. >257.1).

All statistical analyses were conducted using SAS version 9.4 (SAS Institute Inc, Cary, NC, USA). We considered *P* < 0.05 a statistically significant level and used two-sided for all tests in the current analysis.

## RESULTS

We included 100 MASLD patients and 119 healthy controls in the current study. MALSD patients were significantly older than healthy control. The mean (SD) age at enrolment for MALSD cases and controls were 50.0 (15.5) years old and 41.7 (11.7) years old, respectively. The sociodemographic characteristics and selected clinical factors of study participants are presented in Table 1. Compared with healthy controls, MASLD patients were more likely to be male, had higher BMI, more likely to have diabetes and other comorbidity conditions, had lower level of AST but higher level of ALT and GGT. MALSD patients also had higher levels of triglyceride and glucose as well s higher score of FIB-4, kPA, CAP, APRI, NFS (all *P*’s < 0.05). There were no statistically significant differences between MASLD patients and controls with respect to platelet, INR, albumin, cholesterol and BARD score (all *P*’s > 0.05).

Age, BMI, diabetes status, INR, AST, GGT, kPA, CAP score, total bilirubin and APRI were predictors of the MASLD risk in the current study. Accordingly, the ORs and 95% CIs for such factors were: 1.93 (1.00–3.72) (BMI > 23 kg/m^2^ compared with ≤ 23 kg/m^2^); 4.46 (1.16–17.12) (diabetes compared with no diabetes); 2.18 (1.15–4.14) (INR > 1.0 compared with ≤ 1.0); 4.18 (1.54–11.33) (AST≥40U/L compared with < 40U/L); 5.20 (2.39–11.33) (GGT≥55738U/L compared with < 55738U/L); 11.14 (1.29–96.05) (kPA > 8.2 compared with ≤ 8.2); 30.27 (10.07–90.64) (CAP score > 257.1 compared with ≤ 257.1); 1.04 (1.02–1.07) (per age incremental); 1.02 (1.00–1.05) (per unit incremental of total bilirubin); and 49.48 (3.77–649.34 (per unit incremental of APRI score) (Table 2).

## DISCUSSION

In the study of 100 MASLD patients and 119 healthy controls in the Vietnamese population, we identified that while age, BMI, diabetes status, INR, AST, GGT, kPA, CAP score, total bilirubin and APRI were predictors of the MASLD risk, other important factors, such as cardiometabolic condition, platelet, ALT, albumin, cholesterol or triglyceride as well as other non-invasive fibrosis score (i.e., AST/ALT score, NFS, BARD) were not.

To our knowledge, this might be the first study to determine the predictors of MALSD in Vietnamese population. In a recent study of 101 patients with NASH in Ho Chi Minh City, Vietnam, Tran et al.^24^ reported that Fibroscan, APRI an NFS were more accurate than FIB-4 in the diagnostic of patients with F3 or higher. Unfortunately, because there was no control group, it is not possible to evaluate the potential predictors of MASLD in such study population.

While age appeared to be an important determinant of MASLD in our study, it is equally interesting to note that male has significant higher prevalence of MALSD in this population, an observation that is consistent with prior findings,^25^ supporting the hypothesis of endocrine role in the MASLD development (Review in Bertolotti et al.^26^). Accordingly, in males, the prevalence of MASLD tends to increase from younger to middle-aged and to decline at the age of 50 or 60, an “inverted U-shaped” association phenomenon.^26^ Age, on the other hand, is an important risk factor for fibrosis and poor outcomes.^27^

We found that BMI an important predictor or determinant of MALSD in the current study. Noted that the mean and SD of BMI was 23.4 (3.43) in the MASLD patients and 22.1 (2.39) in the control subjects, which can be considered a “lean MASLD population”. A recent study showed that the pooled prevalence of MASLD in lean individuals is about 12% in East Asia, 10% in South Asia, 15.5% in Europe whereas the highest prevalence in Mexico (37.0%) and Italy (26.1%).^28^ It is equally important to point out that about 14% MASLD patients are lean.^29^ A recent meta-analysis, conducted by Ye and colleagues, showed that the pooled incidence of MASLD in lean individuals was 23 per 1,000 person-years.^28^ In a study from Hong Kong, using proton-magnetic resonance spectroscopy to measure changes in liver fat fraction among 565 individuals without MASLD at baseline, Wong et al.^30^ found that the annual incidence of MASLD of 4.3% and an increase in waist circumference, not BMI and plasma triglyceride independent factors that were associated with increased risk of MASLD in lean individuals. In our analysis, triglyceride was one of predictor of MASLD in the univariable regression model, however, the association of triglyceride-MASLD was attenuated in the multivariable regression model.

Similarly, in a recent meta-analysis of 53 studies on 65,029 subjects, including 38,084 lean MASLD and 249,544 healthy subjects, Young et al.^29^ reported that compared to healthy subjects without MASLD, lean MASLD individuals had increased odds for central obesity central obesity (OR = 2.39, 95% CI: 1.75–3.25), hypertension (OR = 2.13, 95% CI: 1.70–2.68), type 2 diabetes mellitus (OR = 3.78, 95% CI: 2.54–5.63), low HDL (OR = 3.09, 95% CI: 1.59–5.99), and metabolic syndrome (OR = 5.85, 95% CI: 4.01–8.63). They also found that lean MALSD patients had higher odds for impaired fasting glucose levels and insulin resistance in a subset of four studies ((OR = 3.06, 95% CI: 2.82–3.32 and OR = 3.99, 95% CI: 2.40–6.61, respectively). Genetically, odds for presence of *PNPLA3* genetic polymorphism of lean MASLD patients, compared with healthy individuals, were almost 3-fold (OR = 2.69, 95% CI: 1.34–5.38). *PNPLA3* is a multifunctional enzyme implicated in the regulation of lipids and retinyl ester activity,^31^ and its variant has been strongly linked to the progression of hepatic fibrosis and an increased risk of HCC.^32–35^ They also found an equally important point that MASLD patients with classic phenotype, compared with lean MALSD patients, had a significantly higher odds of abdominal obesity (OR = 12.10, 95% CI: 8.44–17.35), hypertension (OR = 1.82 (1.53–2.18), type 2 diabetes mellitus (OR = 1.71, 95% CI: 1.34–2.18), impaired fasting glucose (OR = 1.31, 95% CI: 1.18–1.45), low HDL (OR = 1.23, 95% CI: 1.11–1.36), and metabolic syndrome (OR = 3.01, 95% CI: 2.17–4.17).

Another important finding from the current study is that while both kPA and CAP score, critical liver stiffness measurement (LSM) using FibroScan, independent predictors of MALSD, FIB-4 was also found in univariable analysis, and its association was diminished in the multivariable analysis. In a study of 1,040 Indian MASLD patients, De et al.^36^ found that there was no difference in controlled attenuation parameter, FIB-4 score, LSM, FAST score or the proportion of patients in whom advanced fibrosis was ruled-out or ruled-in using FIB-4 or LSM among lean and non-lean MASLD patients. In another study among 911 participants recruited in community in Hong Kong, Wei et al.^37^ reported that compared with obese MASLD patients, non-obese MASLD patients had similar IHTG content but lower cytokeratin-18 fragments and LSM. Another meta-analysis of histologic data showed that compared with non-lean MASLD patients, MASLD patients had lower NAS score, NASH and fibrosis scores,^38^ which is in line with our finding in which we identified NAS score not an independent predictor of MASLD risk in the multivariable regression model.

Pathologically, despite the fact that MASLD patients have lower prevalence of insulin resistance than overweight and obese patients, they have higher prevalence of insulin resistance than healthy individuals without MASLD,^29^ making insulin resistance a major player in the pathogenesis of lean MASLD. An important to note that not all MASLD patients have insulin resistance and multifaceted factors play role in the pathogenesis of MASLD.^39^ Such individuals are lean but metabolically obese. Approximately 20% of individuals with normal body weight have insulin resistance, hypertension or dyslipidemia with the lower prevalence in Caucasians but higher in Asians.^40^ This unique phenotype are described as “metabolically obese normal weight” or MONW, which is associated with significantly higher risk of incident type 2 diabetes mellitus, cardiovascular events and overall mortality, which turns out to be similar outcomes in overweight and obese individuals.^41^

Our study has several limitations. First, because this is a hospital-based case-control study design, selection bias has potentially occurred. Next, residual confounding might also occur even though we used a comprehensive set of covariates in the multivariable regression models. Finally, our results could not be generalizable because the current study was conducted in northern Vietnam.

The present study, however, also has several strengths. To our knowledge, this might be the first study in Vietnamese population that determine the potential predictors of MASLD in a sizable sample. Second, the use of comprehensive set of covariates, including clinical and socioeconomic factors, helped minimize the potential effects of confounding factors.

In summary, in the first study of Vietnamese population, we showed that age, BMI, diabetes status, INR, AST, GGT, kPA, CAP score, total bilirubin and APRI predictors MASLD risk. Findings from our current study lay solid foundation for the construction of risk stratification of MALSD in Vietnamese population. Further studies are thus warranted to replicate our findings that help investigate the underlying mechanisms and construction of risk stratification of MASLD in Asian populations.

## Figures and Tables

**Table 1:** Characteristics of the Metabolic Dysfunction-Associated Steatotic Liver Disease (MALSD) Patients in the Current Study

	Cases (n = 100)	Controls (n = 119)	P-value
Age, Mean (SD)	50.0 (15.5)	41.7 (11.7)	<0.0001
Sex			
Male	61 (61.6)	50 (45.0)	0.02
Female	38 (38.4)	61 (55.0)	
BMI, Mean (SD)	23.4 (3.43)	22.1 (2.39)	0.004
Diabetes			
No	83 (83.0)	116 (97.5)	0.0002
Yes	17 (17.0)	3 (2.5)	
Comorbidity			
No	64 (64.0)	101 (84.9)	0.0004
Yes	36 (36.0)	18 (15.1)	
Platelet	247.1 (69.43)	253.3 (55.82)	0.45
INR, Mean (SD)	2.3 (11.88)	2.9 (13.97)	0.37
AST (U/L), Mean (SD)	37.8 (29.41)	23.7 (9.62)	<0.0001
ALT (U/L), Mean (SD)	45.9 (50.63)	25.2 (20.39)	<0.0001
GGT (U/L), Mean (SD)	77.3 (126.53)	28.9 (25.86)	<0.0001
Albumin (g/L), Mean (SD)	41.4 (4.75)	42.5 (3.80)	0.16
Cholesterol (mmol/L), Mean (SD)	4.9 (1.24)	4.7 (1.10)	0.19
Triglyceride (mmol/L), Mean (SD)	2.0 (1.14)	1.7 (1.66)	0.001
HDL (mmol/L), Mean (SD)	1.3 (1.25)	1.3 (0.35)	0.004
LDL (mmol/L), Mean (SD)	3.1 (1.16)	3.1 (1.73)	0.05
Glucose (mmol/L), Mean (SD)	5.7 (2.07)	4.8 (0.82)	<0.0001
FIB4, Mean (SD)	1.4 (1.29)	0.9 (0.45)	0.0007
kPA, Mean (SD)	8.2 (10.96)	4.1 (1.27)	<0.0001
CAP score, Mean (SD)	257.1 (56.66)	207.7 (41.05)	0.0001
APRI, Mean (SD)	0.4 (0.48)	0.2 (0.12)	**<0.0001**
AST/ALT ratio, Mean (SD)	1.1 (0.55)	1.2 (0.53)	0.004
NFS score. Mean (SD)	−2.3 (1.46)	−2.9 (1.07)	0.002
BARD score. Mean (SD)	1.6 (0.98)	1.7 (0.74)	0.06

**Abbreviations:** ALT: Alanine aminotransferase; AST: Aspartate aminotransferase; BARD: Body mass index (BMI), AST/ALT ratio (AAR), and presence of type 2 diabetes mellitus (DM2); BMI: body mass index; CAP: Controlled attenuation parameter; FIB-4: Fibrosis index based on 4 factors; GGT: Gamma-glutamyl transferase; HDL: High-density lipoprotein; INR: international normalized ratio; kPA: kilopascals; LDL: Low-density Lipoprotein; NFS: nonalcoholic fatty liver disease fibrosis score; SD: standard deviation.

**Table 2:** Predictors of Metabolic Dysfunction-Associated Steatotic Liver Disease (MALSD)

	Cases	Controls	Univariable Model OR (95% CI)	Multivariable Model OR (95% CI)
Sex^a^				
Female	39	66	1.00	1.00
Male	61	53	1.95 (1.13–3.34)	1.32 (0.71–2.42)
BMI^b^				
≤ 23 kg/m^2^	49	80	1.00	1.00
> 23 kg/m^2^	51	39	2.14 (1.23–3.69)	**1.93 (1.00–3.72)**
Diabetes^c^				
No	83	116	1.00	1.00
Yes	17	3	7.92 (2.25–27.89)	**4.46 (1.16–17.12)**
Cardiometabolic condition^d^				
No	8	24	1.00	1.00
Yes	92	95	2.91 (1.24–6.8)	1.11 (0.43–2.89)
Platelet (G/L)^e^				
>200	77	99	1.00	1.00
≤200	23	20	1.48 (0.76–2.89)	1.33 (0.63–2.83)
INR				
≤1.0	45	67	1.00	1.00
>1.0	47	45	1.56 (0.89–2.71)	**2.18 (1.15–4.14)**
AST (U/L)				
<40	74	113	1.00	1.00
≥40	26	6	6.62 (2.60–16.85)	**4.18 (1.54–11.33)**
ALT (U/L)				
<41	70	103	1.00	1.00
≥41	30	16	2.76 (1.40–5.44)	2.06 (0.96–4.42)
GGT (U/L)				
< 55738	52	101	1.00	1.00
≥55738	42	12	6.80 (3.3–14.02)	**5.20 (2.39–11.33)**
Albumin (g/L)				
35–50	89	114	1.00	1.00
<35	11	3	4.70 (1.27–17.34)	3.50 (0.78–15.61)
>50	0	2	--	--
Cholesterol (mmol/L)				
≤5.2	51	79	1.00	1.00
>5.2	44	37	1.84 (1.05–3.23)	**1.76 (0.94–3.29)**
Triglyceride (mmol/L)				
<1.7	45	80	1.00	1.00
≥1.7	51	36	2.52 (1.44–4.42)	1.53 (0.79–2.95)
HDL (mmol/L)				
>0.9	80	105	1.00	1.00
≤0.9	14	11	1.67 (0.72–3.88)	0.90 (0.33–2.42)
LDL (mmol/L)				
<4.0	78	102	1.00	1.00
≥4.0	16	14	1.50 (0.69–3.25)	1.27 (0.54–2.99)
Glucose (mmol/L)				
≤1.0	77	114	1.00	1.00
>1.0	23	5	6.81 (2.48–18.69)	3.23 (0.93–11.19)
HbA1c (%)				
<5.7	20	44	1.00	1.00
≥5.7	26	15	3.81 (1.67–8.71)	1.36 (0.41–4.52)
FIB4				
<1.3	66	105	1.00	1.00
≥1.3	23	14	3.86 (1.93–7.74)	2.03 (0.86–4.82)
kPA				
≤8.2	66	105	1.00	1.00
>8.2	34	14	20.82 (2.7–160.58)	**11.14 (1.29–96.05)**
CAP score				
≤257.1	49	111	1.00	1.00
>257.1	49	7	15.86 (6.71–37.49)	**30.21 (10.07–90.64)**
Age^e^				**1.04 (1.02–1.07)**
Total bilirubin^e^				**1.02 (1.00–1.05)**
APRI^e^				**49.48 (3.77–649.34)**
AST/ALT ratio^e^				0.70 (0.39–1.27)
NFS score^e^				1.00 (0.75–1.39)
BARD score				0.62 (0.43–0.90)

**Abbreviations:** ALT: Alanine aminotransferase; AST: Aspartate aminotransferase; BARD: Body mass index (BMI), AST/ALT ratio (AAR), and presence of type 2 diabetes mellitus (DM2); BMI: body mass index; CAP: Controlled attenuation parameter; FIB-4: Fibrosis index based on 4 factors; GGT: Gamma-glutamyl transferase; HDL: High-density lipoprotein; INR: international normalized ratio; kPA: kilopascals; LDL: Low-density Lipoprotein; NFS: nonalcoholic fatty liver disease fibrosis score; SD: standard deviation.

a Model adjusted for Age, BMI, diabetes, cardio-metabolic condition

b Model adjusted for Age, Sex, Diabetes, cardio-metabolic condition

c Model adjusted for Age, Sex, BMI, cardio-metabolic condition

d Model adjusted for Age, Sex, BMI, Diabetes

e Model adjusted for Age, Sex, BMI, Diabetes, crdio-metabolic codition

## Data Availability

Data of the current study is made available to the corresponding authors based on reasonable request.

## Abbreviations

ALT: Alanine aminotransferase
AST: Aspartate aminotransferase
BARD: Body mass index (BMI), AST/ALT ratio (AAR), and presence of type 2 diabetes mellitus (DM2)
BMI: body mass index
CAP: Controlled attenuation parameter
FIB-4: Fibrosis index based on 4 factors
GGT: Gamma-glutamyl transferase
HDL: High-density lipoprotein
INR: international normalized ratio
kPA: kilopascals
LDL: Low-density Lipoprotein
MASLD: metabolic dysfunction–associated steatotic liver disease
NFS: nonalcoholic fatty liver disease fibrosis score
SD: standard deviation

## References

[1] Bray F, Laversanne M, Sung H, Ferlay J, Siegel RL, Soerjomataram I, Jemal A. Global cancer statistics 2022: GLOBOCAN estimates of incidence and mortality worldwide for 36 cancers in 185 countries. CA Cancer J Clin 2024;74:229–63. 38572751 10.3322/caac.21834

[2] Bray F, Ferlay J, Soerjomataram I, Siegel RL, Torre LA, Jemal A. Global cancer statistics 2018: GLOBOCAN estimates of incidence and mortality worldwide for 36 cancers in 185 countries. CA Cancer J Clin 2018;68:394–424. 30207593 10.3322/caac.21492

[3] Maucort-Boulch D, de Martel C, Franceschi S, Plummer M. Fraction and incidence of liver cancer attributable to hepatitis B and C viruses worldwide. Int J Cancer 2018;142:2471–7. 29388206 10.1002/ijc.31280

[4] Rinella ME, Lazarus JV, Ratziu V, Francque SM, Sanyal AJ, Kanwal F, Romero D, Abdelmalek MF, Anstee QM, Arab JP, Arrese M, Bataller R, A multisociety Delphi consensus statement on new fatty liver disease nomenclature. Hepatology 2023;78:1966–86. 37363821 10.1097/HEP.0000000000000520 PMC10653297

[5] D’Amico G, Garcia-Tsao G, Pagliaro L. Natural history and prognostic indicators of survival in cirrhosis: a systematic review of 118 studies. J Hepatol 2006;44:217–31. 16298014 10.1016/j.jhep.2005.10.013

[6] Younossi Z, Tacke F, Arrese M, Chander Sharma B, Mostafa I, Bugianesi E, Wai-Sun Wong V, Yilmaz Y, George J, Fan J, Vos MB. Global Perspectives on Nonalcoholic Fatty Liver Disease and Nonalcoholic Steatohepatitis. Hepatology 2019;69:2672–82. 30179269 10.1002/hep.30251

[7] Li J, Zou B, Yeo YH, Feng Y, Xie X, Lee DH, Fujii H, Wu Y, Kam LY, Ji F, Li X, Chien N, Prevalence, incidence, and outcome of non-alcoholic fatty liver disease in Asia, 1999–2019: a systematic review and meta-analysis. Lancet Gastroenterol Hepatol 2019;4:389–98. 30902670 10.1016/S2468-1253(19)30039-1

[8] White DL, Kanwal F, El-Serag HB. Association between nonalcoholic fatty liver disease and risk for hepatocellular cancer, based on systematic review. Clin Gastroenterol Hepatol 2012;10:1342–1359.e2. 23041539 10.1016/j.cgh.2012.10.001 PMC3501546

[9] Anstee QM, Reeves HL, Kotsiliti E, Govaere O, Heikenwalder M. From NASH to HCC: current concepts and future challenges. Nat Rev Gastroenterol Hepatol 2019; 10.1038/s41575-019-0145-7 31028350

[10] Anstee QM, Targher G, Day CP. Progression of NAFLD to diabetes mellitus, cardiovascular disease or cirrhosis. Nat Rev Gastroenterol Hepatol 2013;10:330–44. 23507799 10.1038/nrgastro.2013.41

[11] Younossi Z, Anstee QM, Marietti M, Hardy T, Henry L, Eslam M, George J, Bugianesi E. Global burden of NAFLD and NASH: trends, predictions, risk factors and prevention. Nat Rev Gastroenterol Hepatol 2018;15:11–20. 28930295 10.1038/nrgastro.2017.109

[12] Baveno VII - Renewing consensus in portal hypertension - PubMed [Internet]. [cited 2025 Mar 14];Available from: https://pubmed.ncbi.nlm.nih.gov/35120736/

[13] Wai C-T, Greenson JK, Fontana RJ, Kalbfleisch JD, Marrero JA, Conjeevaram HS, Lok AS-F. A simple noninvasive index can predict both significant fibrosis and cirrhosis in patients with chronic hepatitis C. Hepatology 2003;38:518–26. 12883497 10.1053/jhep.2003.50346

[14] Sterling RK, Lissen E, Clumeck N, Sola R, Correa MC, Montaner J, S Sulkowski M, Torriani FJ, Dieterich DT, Thomas DL, Messinger D, Nelson M, Development of a simple noninvasive index to predict significant fibrosis in patients with HIV/HCV coinfection. Hepatology 2006;43:1317–25. 16729309 10.1002/hep.21178

[15] Angulo P, Hui JM, Marchesini G, Bugianesi E, George J, Farrell GC, Enders F, Saksena S, Burt AD, Bida JP, Lindor K, Sanderson SO, The NAFLD fibrosis score: a noninvasive system that identifies liver fibrosis in patients with NAFLD. Hepatology 2007;45:846–54. 17393509 10.1002/hep.21496

[16] Harrison SA, Oliver D, Arnold HL, Gogia S, Neuschwander-Tetri BA. Development and validation of a simple NAFLD clinical scoring system for identifying patients without advanced disease. Gut 2008;57:1441–7. 18390575 10.1136/gut.2007.146019

[17] Xiao G, Zhu S, Xiao X, Yan L, Yang J, Wu G. Comparison of laboratory tests, ultrasound, or magnetic resonance elastography to detect fibrosis in patients with nonalcoholic fatty liver disease: A meta-analysis. Hepatology 2017;66:1486–501. 28586172 10.1002/hep.29302

[18] Sanyal AJ, Foucquier J, Younossi ZM, Harrison SA, Newsome PN, Chan W-K, Yilmaz Y, De Ledinghen V, Costentin C, Zheng M-H, Wai-Sun Wong V, Elkhashab M, Enhanced diagnosis of advanced fibrosis and cirrhosis in individuals with NAFLD using FibroScan-based Agile scores. J Hepatol 2023;78:247–59. 36375686 10.1016/j.jhep.2022.10.034 PMC10170177

[19] Lin H, Lee HW, Yip TC-F, Tsochatzis E, Petta S, Bugianesi E, Yoneda M, Zheng M-H, Hagström H, Boursier J, Calleja JL, Goh GB-B, Vibration-Controlled Transient Elastography Scores to Predict Liver-Related Events in Steatotic Liver Disease. JAMA 2024;331:1287–97. 38512249 10.1001/jama.2024.1447 PMC10958386

[20] Kamali L, Adibi A, Ebrahimian S, Jafari F, Sharifi M. Diagnostic Performance of Ultrasonography in Detecting Fatty Liver Disease in Comparison with Fibroscan in People Suspected of Fatty Liver. Adv Biomed Res 2019;8:69. 31897407 10.4103/abr.abr_114_19 PMC6909544

[21] Ziol M, Handra-Luca A, Kettaneh A, Christidis C, Mal F, Kazemi F, de Lédinghen V, Marcellin P, Dhumeaux D, Trinchet J-C, Beaugrand M. Noninvasive assessment of liver fibrosis by measurement of stiffness in patients with chronic hepatitis C. Hepatology 2005;41:48–54. 15690481 10.1002/hep.20506

[22] Shah AG, Lydecker A, Murray K, Tetri BN, Contos MJ, Sanyal AJ, Nash Clinical Research Network. Comparison of noninvasive markers of fibrosis in patients with nonalcoholic fatty liver disease. Clin Gastroenterol Hepatol 2009;7:1104–12. 19523535 10.1016/j.cgh.2009.05.033 PMC3079239

[23] Sumida Y, Yoneda M, Hyogo H, Itoh Y, Ono M, Fujii H, Eguchi Y, Suzuki Y, Aoki N, Kanemasa K, Fujita K, Chayama K, Validation of the FIB4 index in a Japanese nonalcoholic fatty liver disease population. BMC Gastroenterol 2012;12:2. 22221544 10.1186/1471-230X-12-2 PMC3266187

[24] Tran TK, Nguyen DM. The role of non-invasive methods in evaluating liver fibrosis of patients with no-alcoholic steatohepatitis. Imaging Medicine 2018;10:159–63.

[25] Lonardo A, Carani C, Carulli N, Loria P. “Endocrine NAFLD” a hormonocentric perspective of nonalcoholic fatty liver disease pathogenesis. J Hepatol 2006;44:1196–207. 16618516 10.1016/j.jhep.2006.03.005

[26] Bertolotti M, Lonardo A, Mussi C, Baldelli E, Pellegrini E, Ballestri S, Romagnoli D, Loria P. Nonalcoholic fatty liver disease and aging: epidemiology to management. World J Gastroenterol 2014;20:14185–204. 25339806 10.3748/wjg.v20.i39.14185 PMC4202348

[27] Meeuwsen S, Horgan GW, Elia M. The relationship between BMI and percent body fat, measured by bioelectrical impedance, in a large adult sample is curvilinear and influenced by age and sex. Clin Nutr 2010;29:560–6. 20359792 10.1016/j.clnu.2009.12.011

[28] Ye Q, Zou B, Yeo YH, Li J, Huang DQ, Wu Y, Yang H, Liu C, Kam LY, Tan XXE, Chien N, Trinh S, Global prevalence, incidence, and outcomes of non-obese or lean non-alcoholic fatty liver disease: a systematic review and meta-analysis. Lancet Gastroenterol Hepatol 2020;5:739–52. 32413340 10.1016/S2468-1253(20)30077-7

[29] Young S, Tariq R, Provenza J, Satapathy SK, Faisal K, Choudhry A, Friedman SL, Singal AK. Prevalence and Profile of Nonalcoholic Fatty Liver Disease in Lean Adults: Systematic Review and Meta-Analysis. Hepatol Commun 2020;4:953–72. 32626829 10.1002/hep4.1519 PMC7327210

[30] Wong VW-S, Wong GL-H, Yeung DK-W, Lau TK-T, Chan CK-M, Chim AM-L, Abrigo JM, Chan RS-M, Woo J, Tse Y-K, Chu WC-W, Chan HL-Y. Incidence of non-alcoholic fatty liver disease in Hong Kong: a population study with paired proton-magnetic resonance spectroscopy. J Hepatol 2015;62:182–9. 25195550 10.1016/j.jhep.2014.08.041

[31] Bruschi FV, Claudel T, Tardelli M, Caligiuri A, Stulnig TM, Marra F, Trauner M. The PNPLA3 I148M variant modulates the fibrogenic phenotype of human hepatic stellate cells. Hepatology 2017;65:1875–90. 28073161 10.1002/hep.29041

[32] Stender S, Loomba R. PNPLA3 Genotype and Risk of Liver and All-Cause Mortality. Hepatology 2020;71:777–9. 31954067 10.1002/hep.31113 PMC7979358

[33] Singal AG, Manjunath H, Yopp AC, Beg MS, Marrero JA, Gopal P, Waljee AK. The effect of PNPLA3 on fibrosis progression and development of hepatocellular carcinoma: a meta-analysis. Am J Gastroenterol 2014;109:325–34. 24445574 10.1038/ajg.2013.476 PMC5610907

[34] Stickel F, Buch S, Nischalke HD, Weiss KH, Gotthardt D, Fischer J, Rosendahl J, Marot A, Elamly M, Casper M, Lammert F, McQuillin A, Genetic variants in PNPLA3 and TM6SF2 predispose to the development of hepatocellular carcinoma in individuals with alcohol-related cirrhosis. Am J Gastroenterol 2018;113:1475–83. 29535416 10.1038/s41395-018-0041-8

[35] Buch S, Stickel F, Trépo E, Way M, Herrmann A, Nischalke HD, Brosch M, Rosendahl J, Berg T, Ridinger M, Rietschel M, McQuillin A, A genome-wide association study confirms PNPLA3 and identifies TM6SF2 and MBOAT7 as risk loci for alcohol-related cirrhosis. Nat Genet 2015;47:1443–8. 26482880 10.1038/ng.3417

[36] De A, Mehta M, Singh P, Bhagat N, Mitra S, Das A, Duseja A. Lean Indian patients with non-alcoholic fatty liver disease (NAFLD) have less metabolic risk factors but similar liver disease severity as non-lean patients with NAFLD. Int J Obes (Lond) 2023;47:986–92. 37474570 10.1038/s41366-023-01346-w

[37] Wei JL, Leung JC-F, Loong TC-W, Wong GL-H, Yeung DK-W, Chan RS-M, Chan HL-Y, Chim AM-L, Woo J, Chu WC-W, Wong VW-S. Prevalence and Severity of Nonalcoholic Fatty Liver Disease in Non-Obese Patients: A Population Study Using Proton-Magnetic Resonance Spectroscopy. Am J Gastroenterol 2015;110:1306–14; quiz 1315. 26215532 10.1038/ajg.2015.235

[38] Sookoian S, Pirola CJ. Systematic review with meta-analysis: the significance of histological disease severity in lean patients with nonalcoholic fatty liver disease. Aliment Pharmacol Ther 2018;47:16–25. 29083036 10.1111/apt.14401

[39] Singh SP, Misra B, Kar SK, Panigrahi MK, Misra D, Bhuyan P, Pattnaik K, Meher C, Agrawal O, Rout N, Swain M. Nonalcoholic fatty liver disease (NAFLD) without insulin resistance: Is it different? Clin Res Hepatol Gastroenterol 2015;39:482–8. 25543522 10.1016/j.clinre.2014.08.014

[40] Wang B, Zhuang R, Luo X, Yin L, Pang C, Feng T, You H, Zhai Y, Ren Y, Zhang L, Li L, Zhao J, Prevalence of Metabolically Healthy Obese and Metabolically Obese but Normal Weight in Adults Worldwide: A Meta-Analysis. Horm Metab Res 2015;47:839–45. 26340705 10.1055/s-0035-1559767

[41] Kramer CK, Zinman B, Retnakaran R. Are metabolically healthy overweight and obesity benign conditions?: A systematic review and meta-analysis. Ann Intern Med 2013;159:758–69. 24297192 10.7326/0003-4819-159-11-201312030-00008

